# Lifestyle Characteristics and Gastroesophageal Reflux Disease: A Population-Based Study in Albania

**DOI:** 10.1155/2013/936792

**Published:** 2013-02-24

**Authors:** Lulzim Çela, Bledar Kraja, Kliti Hoti, Ervin Toçi, Herion Muja, Enver Roshi, Genc Burazeri

**Affiliations:** ^1^Department of Public Health, Faculty of Medicine, Tirana University, Tirana, Albania; ^2^Service of Gastro-Hepatology, Mother Theresa University Hospital Center, Rr. “Dibrës”, No. 371, Tirana, Albania; ^3^Department of International Health, Faculty of Health, Medicine and Life Sciences, Maastricht University, Maastricht, The Netherlands

## Abstract

*Aim*. We aimed to assess the prevalence and lifestyle correlates of gastroesophageal reflux disease (GERD) in the adult population of Albania, a Mediterranean country in Southeast Europe which has experienced major behavioral changes in the past two decades. *Methods*. A cross-sectional study, conducted in 2012, included a population-representative sample of 845 individuals (≥18 years) residing in Tirana (345 men, mean age: 51.3 ± 18.5; 500 women, mean age: 49.7 ± 18.8; response rate: 84.5%). Assessment of GERD was based on Montreal definition. Covariates included socioeconomic characteristics, lifestyle factors, and body mass index. Logistic regression was used to assess the association of socioeconomic characteristics and lifestyle factors with GERD. *Results*. The overall prevalence of GERD was 11.9%. There were no significant sex differences, but a higher prevalence among the older participants. In fully adjusted models, there was a positive relationship of GERD with smoking, physical inactivity, fried food consumption, and obesity, but not so for alcohol intake and meat consumption. *Conclusion*. We obtained important evidence on the prevalence and lifestyle correlates of GERD in a Western Balkans' country. Smoking, physical inactivity, and obesity were strong “predictors” of GERD in this population. Findings from this study should be replicated in prospective studies in Albania and other transitional settings.

## 1. Introduction 

Gastroesophageal reflux disease (GERD) is a condition usually manifesting such symptoms as heartburn and acid regurgitation [1]. Nevertheless, symptoms may also include chest pain or evidence of extra-esophageal manifestations such as pulmonary, ear, nose, or throat symptoms. Most of the patients have no visible mucosal damage at the time of endoscopy (nonerosive GERD), whereas a few others may have esophagitis, peptic strictures, Barrett's esophagus, or esophageal adenocarcinoma [2].

GERD is a multifactorial process and one of the most common diseases. Causes of GERD are not clear, although it is recognized that increased transient lower esophageal sphincter relaxations and the presence of significant hiatal hernia contribute to development of the disease [2]. Typically, GERD begins in the middle age, suggesting that various environmental and lifestyle factors may contribute to its pathophysiology. Dietary factors such as shorter dinner-to-bed time, a high dietary fat intake, obesity, and smoking have been implicated in increasing the risk for GERD. Other lifestyle factors include stress, major negative life events, and alcoholism [2–5]. Furthermore, residents in rural areas and those with a positive family history are associated with a higher risk of GERD [6, 7]. Socioeconomic status and a “westernized” diet, suggested as potential risk factors, have not been confirmed yet.

Estimates of the actual prevalence of GERD are difficult to obtain, because individuals seeking health care probably represent only the tip of the iceberg. The epidemiology of GERD is primarily based on population-based surveys conducted in the affluent western regions like the United States and Europe [6, 8]. In different studies, 10–20% of the adult western population has been reported to experience GERD symptoms (heartburn and/or regurgitation) at least once per week [9]. 

Population-based data on the magnitude and distribution of GERD in transitional countries of Southeast Europe including Albania are scarce. Traditionally, Albanian diet has been characterized by a low consumption of total energy, meat, and dairy products, but a high consumption of fruit, vegetables, and carbohydrates [10]. However, after the breakdown of the communist regime in early 1990s, Albania has undergone a rapid transition including dietary changes [11] with an emergent “western” behavior consisting of processed foods higher in salt and saturated fats. 

In this context, we aimed to assess the prevalence and socioeconomic and lifestyle correlates of GERD in the adult population of Albania, a country which has experienced major dietary changes in the past decades in line with the socioeconomic and political transition towards an open-market system. 

## 2. Material and Methods

A cross-sectional study was conducted in Tirana, the Albanian capital, in March–August, 2012.

### 2.1. Study Population

A simple random sample of 1000 adult individuals (≥18 years) was drawn based on family physicians' lists in Tirana municipality. Calculations of the sample size were made for a number of behavioral hypotheses including smoking, physical inactivity, and dietary patterns. The significance level (two-tailed) was taken as 5% and the power was set at 80%. Based on conservative calculations, the required minimal number of participants to be recruited was about 700. We decided to recruit 1000 individuals in order to increase the power of the study considering also the potential degree of non-response. 

Of 1000 people in the sample, 845 individuals agreed to participate and were subsequently examined at primary health care centers in Tirana (345 men, mean age: 51.3 ± 18.5; 500 women, mean age: 49.7 ± 18.8; overall response rate: 845/1000 = 84.5%). 

### 2.2. Data Collection

The Montreal instrument for assessment of GERD [1] was translated into the Albanian language by following the standard methods of cross-cultural adaptation of the questionnaires. Subsequently, the Albanian version of the tool was pretested in a small sample of individuals (*N* = 30) attending primary health care services in Tirana before conducting the current survey.

In line with the Montreal definition of GERD for population-based studies [1], individuals were classified into two groups based on the presence (or absence) of GERD. Participants were considered as having GERD if, during last year, they reported heartburn or regurgitation occurring at least once a week and having at least moderate problems from such symptoms. Participants reporting use of medications for heartburn or regurgitation at least once weekly were also included in the GERD group, irrespective of symptom severity. Heartburn was defined as a burning sensation in the retrosternal area (behind the breastbone). Regurgitation was defined as the perception of flow of refluxed gastric content into the mouth or hypopharynx. Conversely, individuals with no reflux symptoms and/or those with reflux symptoms that were not regarded troublesome were classified as non-GERD.

In addition, participants were asked about their smoking habits (categorized into: current smoker, former smoker, and never smoker), alcohol intake (dichotomized into: no/occasional intake versus moderate/heavy intake), physical exercise (low, moderate, and high), and dietary habits including frequency of meat consumption and frequency of fried food consumption (each trichotomized into: frequent consumption, moderate consumption, and rare/no consumption).

Demographic (age and sex) and socioeconomic data (educational attainment (years of formal schooling, trichotomized subsequently into: 0–8 years, 9–12 years, ≥13 years) and income level (low, middle, and high)) were also collected. 

Physical examination included measurement of height and weight, based on which, body mass index (BMI: kg/m^2^) was calculated for each participant. 

### 2.3. Statistical Analysis

Binary logistic regression was used to assess the association of demographic and socioeconomic characteristics and behavioral/lifestyle factors with GERD. Initial models were unadjusted. Next models included adjustment for age. Subsequently, logistic models were additionally adjusted simultaneously for sex and socioeconomic factors (education and income level). Finally, lifestyle factors (smoking, alcohol intake, physical activity, frequency of meat consumption, and frequency of  fried food consumption) and BMI were also introduced into the logistic models in a backward stepwise elimination procedure with a *P* value to exit set at *P* < 0.10. Unadjusted (crude), next age-adjusted, and finally multivariable-adjusted odds ratios (ORs) and their respective 95% confidence intervals (95% CIs) were calculated. Hosmer-Lemeshow goodness-of-fit test was used to assess the validity of the logistic models; all models satisfied the criterion. All the statistical analysis was conducted in SPSS (Statistical Package for Social Sciences, version 15.0).

## 3. Results

The overall prevalence of GERD in this study population was 101/845 = 11.9%. There were no significant sex differences, but a higher prevalence among the older participants (>40 years) compared with their younger counterparts (≤40 years) (*P* = 0.015, Table 1). Furthermore, there was evidence of an inverse linear relationship of GERD with age (overall *P* = 0.018), but no association with income level.

The prevalence of GERD was substantially and significantly higher among current smokers (*P* < 0.001), individuals who reported low levels of physical exercise (*P* = 0.004), those with moderate-to-heavy alcohol intake (*P* = 0.006), participants who reported a high frequency of meat and/or fried food consumption (*P* = 0.091 and *P* < 0.001, resp.), and among obese individuals (*P* < 0.001) (Table 2). 

In age-adjusted logistic regression models, smoking, alcohol intake, physical inactivity, frequent consumption of meat and/or fried food, and obesity were all positively and significantly associated with GERD (Table 3, model 1). Upon further adjustment for sex and income level, there was evidence of an inverse association of GERD with educational attainment (Table 3, model 2), whereas the strong relationships with each lifestyle characteristic and obesity persisted strongly after additional adjustment for sex and socioeconomic variables (Table 3, model 2). In fully-adjusted models with all covariates included in a backward stepwise elimination procedure, the positive relationship of GERD with smoking, physical inactivity, and fried food consumption persisted strongly and significantly, but not so for alcohol intake and meat consumption (Table 3, model 3). In addition, upon multivariable-adjustment, there was evidence of a positive relationship of GERD with age (Table 3, model 3). 

## 4. Discussion

To the best of our knowledge, this is the first study assessing the prevalence and distribution of GERD in Albania: a Mediterranean country in Southeast Europe. Findings from this study indicate that the prevalence of GERD in Albania is about 12%, and that smoking, physical inactivity, a frequent consumption of fried food, and obesity were all positively and significantly associated with GERD.

The prevalence of GERD in Albania seems lower than that observed in other (mainly Western) populations [9]. It should be noted that the prevalence of GERD has been increasing in Western countries over the past 30 years [9]. It has been argued that the decreasing prevalence of Helicobacter pylori may play an important role to the increasing prevalence of GERD in these regions. Recent data suggests that many patients with Helicobacter pylori-induced gastritis have involvement of the antrum and corpus, decreasing parietal cell mass, reducing acid secretion, and elevating gastric pH. This may have a protective effect on the esophageal mucosa in patients susceptible to GERD [12]. Furthermore, in Asian populations, other possible reasons for the lower GERD prevalence include low dietary fat intake, low BMI, and a lower gastric acid output, possibly related to Helicobacter pylori infection [13]. Our findings are comparable with reports from countries with a high prevalence of Helicobacter pylori infection [13]. Therefore, the seemingly lower prevalence of GERD in our study population may be related to the high prevalence of Helicobacter pylori infection in the Albanian population [14]. 

On the other hand, the different age ranges examined in various studies might explain the differing results between studies involving assessment of GERD. In our study, there was evidence of a positive relationship of GERD with age. However, the relationship between GERD and age is controversial in that some studies have observed a positive relationship [15, 16], a few other studies have reported an inverse association [17, 18], whereas some further reports have pointed to a lack of association between age and GERD [19, 20]. In our study, in fully-adjusted models, the positive association of age with GERD was borderline significant, similar to a prior report from Locke et al. [21]. 

In our study, obesity was strongly and significantly associated with GERD, in line with the findings observed in other populations [21, 22], but in contrast with findings reported by Lagergren et al. [23]. A positive relationship between BMI and symptomatic GERD was previously observed in a sample of adult patients in Albania where, upon adjustment for socioeconomic characteristics and behavioral factors, the positive association of GERD with BMI persisted strongly [24]. A potential biological mechanism underlying an increased risk of reflux among obese persons has been suggested through increased extrinsic gastric compression by surrounding adipose tissue and anatomical disruption of the gastroesophageal junction [8, 25]. Our study assessed a wide range of behavioral/lifestyle factors associated with GERD in the Albanian adult population. Frequent consumption of meat and/or fried food, smoking, and physical inactivity were all positively associated with GERD. Consumption of meat was confirmed as a risk factor for GERD in this Albanian population (notwithstanding the lack of statistical significance in fully-adjusted logistic regression models due to multi-collinearity with other covariates, mainly with fried food consumption), compatible with results from other studies conducted elsewhere [26]. Higher fat content of meat may be responsible for this increased risk, as fat delays gastric emptying and, therefore, meat is a well-accepted risk factor for GERD [26].

The relationship of GERD with cigarette smoking has been reported in different studies [6, 21]. Three cross sectional studies have indicated a significant positive association between GERD symptoms and smoking, in the same line with our findings [6, 21]. Furthermore, a few longitudinal studies have investigated the relationship with smoking. The UK-GP database study found that there were significantly more former smokers (OR 1.2, 95% CI = 1.1–1.4) and slightly more current smokers (OR 1.1, 95% CI = 1.0–1.2) among patients with a new diagnosis of GERD than in the control cohort [4]. 

In our study, upon multivariable adjustment, there was no significant relationship with alcohol intake. Previous studies provide inconsistent results with no association of GERD with alcohol consumption [4, 9] (consistent with our findings), whereas a study conducted in Germany showed alcohol to be a significant risk factor [26].

We observed a strong inverse relationship between physical activity and GERD which, on the face of it, is counterintuitive. However, previous studies have indicated conflicting results with regard to physical exercise [27, 28]. 

A fairly recent study concluded that intermediate frequency of physical activity might decrease the risk of GERD among obese individuals, while no influence of physical activity on GERD was found in non-obese participants [29]. 

Conversely, it has been argued that physical exercise may increase the risk of GERD possibly by increasing transient relaxation of the lower esophageal sphincter, or by decreasing the gastrointestinal blood flow and changing the esophageal and gastric motor function [30–32]. A plausible explanation for the strong positive relationship between physical inactivity and GERD in our study may be the avoidance of physical exercise among people with GERD symptoms.

A major strength of the current study conducted in Albania includes the population-based design involving a representative sample of adult men and women with a high participation rate (84.5%). In our study, all symptoms were measured with a well-validated instrument fulfilling the consensus criteria for GERD [1]. Nevertheless, we cannot fully dismiss the possibility of information bias. More importantly, findings from cross-sectional studies are not assumed to be causal. From this point of view, the issue of reverse causality, including lifestyle modification (e.g., changes in dietary patterns and/or physical exercise) after onset of GERD symptoms, remains unresolved from such cross-sectional designs.

## 5. Conclusions

We obtained important evidence on the prevalence and lifestyle correlates of GERD in the adult population of Albania, a transitional country in the Western Balkans which, in the past two decades, has experienced a major deviation from the traditional Mediterranean diet to a “western” behavioral prototype including intake of junk food and processed food. However, findings from this study should be replicated in prospective studies in Albania and other transitional settings.

## Figures and Tables

**Table 1:** Distribution of gastro esophageal reflux disease (GERD) by demographic and socioeconomic characteristics of a representative sample of Albanian adults.

Variable	No GERD	GERD	OR (95% CI)^c^	*P* value
Sex				
Men	298 (86.4)^a^	47 (13.6)	1.30 (0.86–1.98)	0.215
Women	446 (89.2)	54 (10.8)	1.00 (reference)	
Age (years)	52.0 (32.0)^b^	57.0 (25.0)	1.02 (1.01–1.03)	0.001
Age				
≤40 years	240 (91.6)	22 (8.4)	1.00 (reference)	0.015
>40 years	465 (85.5)	79 (14.5)	1.85 (1.13–3.05)	
Educational level (years)	12.0 (4.0)^b^	12.0 (6.75)	0.92 (0.87–0.98)	0.003
Educational level				**0.018 (2)** ^ d^
Low (0–8) years	111 (81.6)	25 (18.4)	2.23 (1.27–3.93)	0.005
Middle (9–12) years	273 (86.4)	43 (13.6)	1.56 (0.96–2.53)	0.072
High (≥13 years)	317 (90.8)	32 (9.2)	1.00 (reference)	—
Income level				**0.474 (2)**
Low	83 (84.7)	15 (15.3)	1.57 (0.75–3.27)	0.232
Middle	450 (87.0)	67 (13.0)	1.29 (0.74–2.24)	0.365
High	156 (89.7)	18 (10.3)	1.00 (reference)	—

^a^Numbers and row percentages (in parentheses). Discrepancies in the totals are due to missing covariate values.

^b^Median and interquartile range (in parentheses).

^c^Unadjusted (crude) odds ratios (ORs: GERD versus no GERD), 95% confidence intervals (95% CIs), and *P* values from binary logistic regression.

^d^Overall *P* value and degrees of freedom (in parentheses).

**Table 2:** Distribution of lifestyle characteristics of participants with and without gastro esophageal reflux disease (GERD).

Variable	No GERD	GERD	OR (95% CI)^b^	*P* value
Smoking				**<0.001 (2)** ^ c^
Current smoker	129 (67.9)^a^	61 (32.1)	12.3 (7.17–21.15)	<0.001
Former smoker	75 (78.9)	20 (21.1)	6.95 (3.57–13.5)	<0.001
Never smoker	521 (96.3)	20 (3.7)	1.00 (reference)	—
Alcohol consumption				
No/Occasional intake	539 (89.4)	64 (10.6)	1.00 (reference)	0.006
Moderate/Heavy intake	152 (81.7)	34 (18.3)	1.88 (1.20–2.96)	
Physical activity				**<0.001 (2)**
Low	150 (78.9)	40 (21.1)	6.32 (2.75–14.54)	0.004
Moderate	370 (53.9)	51 (12.1)	3.27 (1.45–7.36)	<0.001
High	166 (96.0)	7 (4.0)	1.00 (reference)	—
Fried food frequency				**<0.001 (2)**
Frequent consumption	266 (81.8)	59 (18.2)	3.13 (1.86–5.91)	<0.001
Moderate consumption	224 (90.0)	25 (10.0)	1.67 (0.87–3.20)	0.125
Rare/no consumption	239 (93.7)	16 (6.3)	1.00 (reference)	—
Meat consumption frequency				**0.050 (2)**
Frequent consumption	548 (85.9)	90 (14.1)	3.45 (0.82–14.50)	0.091
Moderate consumption	99 (92.5)	8 (7.5)	1.70 (0.35–8.33)	0.515
Rare/no consumption	42 (95.5)	2 (4.5)	1.00 (reference)	—
BMI (kg/m^2^)	26.2 (5.48)^d^	28.7 (5.91)	1.08 (1.04–1.13)	<0.001
BMI				**<0.001 (2)**
<25	265 (93.3)	19 (6.7)	1.00 (reference)	—
25–29.9	306 (87.9)	42 (12.1)	1.91 (1.09–3.37)	0.025
≥30	128 (76.2)	40 (23.8)	4.36 (2.43–7.83)	<0.001

^a^Numbers and row percentages (in parentheses). Discrepancies in the totals are due to missing covariate values.

^b^Unadjusted (crude) odds ratios (ORs: GERD versus no GERD), 95% confidence intervals (95% CIs), and *P* values from binary logistic regression.

^c^Overall *P* value and degrees of freedom (in parentheses).

^d^Median and interquartile range (in parentheses).

**Table 3:** Association of demographic, socioeconomic, and lifestyle factors with GERD; age-adjusted and multivariable-adjusted odds ratios (ORs) from binary logistic regression.

Variable	Model 1^a^		Model 2^b^		Model 3^c^	
	OR (95% CI)	*P *	OR (95% CI)	*P *	OR (95% CI)	*P *
Sex						
Men Women	1.27 (0.83–1.94) 1.00 (reference)	0.270	1.36 (0.88–2.10) 1.00 (reference)	0.168	0.32 (0.17–0.59) 1.00 (reference)	<0.001
Age ≤40 years >40 years	—	—	1.00 (reference) 1.56 (0.92–2.65)	0.099	1.00 (reference) 1.89 (0.96–3.76)	0.067
Educational level		**0.147 (2)** ^ d^		**0.063 (2)**		
Low (0–8) years	1.94 (1.08–3.51)	0.028	2.06 (1.12–3.77)	0.020		
Middle (9–12) years	1.42 (0.87–2.33)	0.162	1.45 (0.87–2.39)	0.151		
High (≥13 years)	1.00 (reference)	—	1.00 (reference)	—		
Income level		**0.655 (2)**		**0.854 (2)**		
Low	1.41 (0.67–2.96)	0.371	1.22 (0.57–2.59)	0.612		
Middle	1.22 (0.70–2.13)	0.483	1.15 (0.66–2.02)	0.622		
High	1.00 (reference)	—	1.00 (reference)	—		
BMI		**<0.001 (2)**		**<0.001 (2)**		**0.002 (2)**
<25	1.00 (reference)	—	1.00 (reference)	—	1.00 (reference)	—
25–29.9	1.79 (1.00–3.21)	0.050	1.58 (0.87–2.85)	0.130	1.71 (0.86–3.38)	0.124
≥30	3.97 (2.12–7.41)	<0.001	3.74 (1.99–7.01)	<0.001	3.79 (1.79–8.03)	<0.001
Smoking		**<0.001 (2)**		**<0.001 (2)**		**<0.001 (2)**
Current smoker	13.36 (7.7–23.2)	<0.001	23.6 (12.3–45.3)	<0.001	29.3 (13.9–61.2)	<0.001
Former smoker	6.03 (3.06–11.8)	<0.001	12.68 (5.9–27.2)	<0.001	9.79 (4.22–22.7)	<0.001
Never smoker	1.00 (reference)	—	1.00 (reference)	—	1.00 (reference)	—
Alcohol consumption						
No/Occasional intake Moderate/Heavy intake	1.00 (reference) 1.79 (1.13–2.83)	0.013	1.00 (reference) 1.83 (1.10–3.06)	0.021		
Physical activity		**<0.001(2)**		**<0.001 (2)**		**<0.001 (2)**
Low	5.79 (2.46–13.6)	<0.001	5.47 (2.32–12.9)	<0.001	7.03 (2.68–18.4)	<0.001
Moderate	3.10 (1.37–7.01)	0.007	3.01 (1.33–6.84)	0.008	2.75 (1.11–6.78)	0.028
High	1.00 (reference)	—	1.00 (reference)	—	1.00 (reference)	—
Fried food frequency		**<0.001 (2)**		**<0.001 (2)**		**0.001 (2)**
Frequent	4.13 (2.28–7.48)	<0.001	3.57 (1.97–6.49)	<0.001	3.01 (1.52–6.20)	0.002
Moderate	1.91 (0.99–3.69)	0.055	1.69 (0.86–3.32)	0.128	1.21 (0.55–2.65)	0.634
Not frequent	1.00 (reference)	—	1.00 (reference)	—	1.00 (reference)	—
Meat consumption frequency		**0.057 (2)**		**0.060 (2)**		
Frequent	3.52 (0.83–14.8)	0.087	3.93 (0.91–17.0)	0.068		
Moderate	1.79 (0.36–8.81)	0.476	2.09 (0.41–10.6)	0.372		
Not frequent	1.00 (reference)	—	1.00 (reference)	—		

^a^Model 1: adjusted for age.

^b^Model 2: simultaneously adjusted for age, sex, and socioeconomic variables (education and income level).

^c^Model 3: adjusted for age, sex, socioeconomic variables, and behavioral factors (smoking, alcohol intake, physical activity, frequency of meat consumption, frequency of fired food consumption, and body mass index). All variables were entered in a backward stepwise elimination procedure with a *P* value to exit set at <0.10. Empty cells refer to the variables excluded from the final model.

^d^Overall *P* value and degrees of freedom (in parentheses).
